# Supplementary material for: An assessment of the immune costs associated with meiotic drive elements in Drosophila
Source: Proc Biol Sci. 2019 Sep 18;286(1911):20191534. doi: 10.1098/rspb.2019.1534 (PMC6784720; doi:10.1098/rspb.2019.1534)
Supplement: Supplementary Figure 1 [file rspb20191534supp2.pdf]

Supplemental Figures:

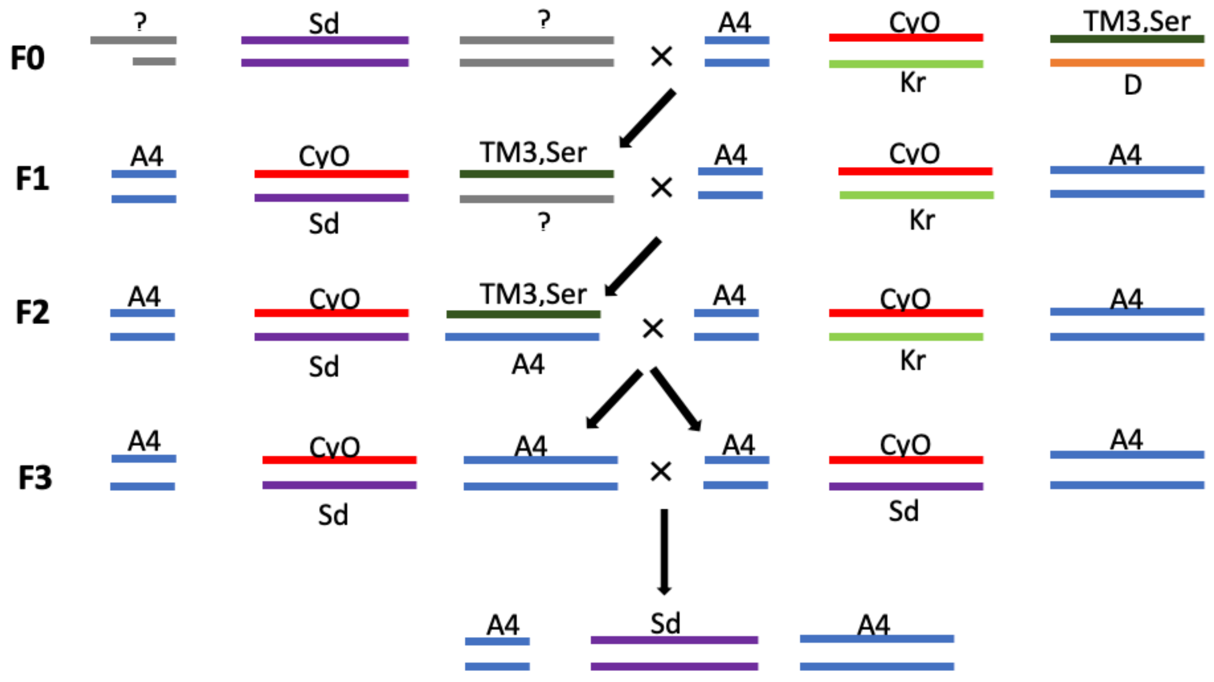

Figure S1. Crossing scheme to generate *D. melanogaster* lines with isogenic backgrounds. In order to move the Sd 2nd chromosome into an A4 background males from the original Sd and control Zambia lines were crossed to female virgins from lines containing balancers that produced mutant phenotypes and to sisters in the final cross. In F0 the original lines' males (F0, left) were crossed to female virgins with double balancers on the 2nd and 3rd chromosome and an A4 X (F0, right). The resulting normal-eyed, curly and serrated winged males sired from the F0 cross carrying the A4 X (F1, left) were crossed to virgin females with fixed A4 X and 3rd chromosome (F1, right). Normal-eyed, curly and serrated winged male progeny heterozygous for A4 on the 3rd chromosome (F2, left) were then backcrossed to their mothers (F2, right) to clean up the 3rd chromosome. This produced normal-eyed, curly winged males fixed with A4 X and 3rd chromosome and heterozygous for Sd on their 2nd (F3, left) which were crossed to their sisters carrying the same genetic background (F3, right). Finally, straight

winged normal-eyed progeny, with exception for Sd homozygous lethal lines maintained over the Cyo balancer, were selected for and crossed to each other to maintain lines with fixed A4 X, A4 3rd chromosome, and homozygous Sd 2nd chromosome.
